# Family presence during invasive procedures: a pilot study to test a tool

**DOI:** 10.1186/s12913-022-08876-5

**Published:** 2022-12-26

**Authors:** Eva de Mingo-Fernández, Ángel Belzunegui-Eraso, Guillermina Medina-Martín, Roser Cuesta-Martínez, Raquel Tejada-Musté, María Jiménez-Herrera

**Affiliations:** 1 Departament d’Infermeria (Nursing Department), Universitat Rovira i Virgili, Tarragona, Spain; 2 Consorci Sanitari del l’Alt Penedès i Garraf. (CSAPG), Barcelona, Spain; 3 Departament estadística (Statistics Department), Universitat Rovira i Virgili, Tarragona, Spain

**Keywords:** Attitude of health personnel, Family, Invasive procedures, Ethic, Professional-family relations

## Abstract

**Background:**

Family Presence During Invasive Procedures (FPDI) generates controversy among healthcare professionals. Twibell and her team designed an instrument that measured nurses’ Risk-Benefit and Self-Confidence perceptions regarding family presence during resuscitation and was used in numerous studies.

**Objectives:**

Evaluate the new tool for Family Presence Risk-Benefit and Family Presence Self-Confidence during invasive procedures and find out the opinions of the medical and nursing staff on FPDIP.

**Method:**

Cross-sectional methodological pilot study. Online and paper questionnaires modified from a previous translation. A factor analysis was performed for the validity of the indices and bivariate analysis for all the variables. Ethical approvals and research permissions were obtained according to national standards.

**Results:**

One hundred twenty healthcare professionals (22.18%) answered the survey. Cronbach’s α on the Family Presence Risk-Benefit scale was 0.877. Cronbach’s α on the Family Presence Self-Confidence scale was 0.937. The correlation between the Risk-Benefit and Self-confidence variables is significant and with a moderate intensity of the relationship. A lower predisposition to Family Presence During Invasive Procedures is observed. Physicians are more reluctant than nurses.

**Conclusions:**

The FPDI generates controversy as it alters health professionals’ routines when they decide whether to allow it or not. There is a tendency for younger professionals to support FPDI. In general, health professionals, mainly physicians, do not favor FPDI. Health workers who perceive fewer risks and more benefits in FPDI and have greater self-confidence are more in favor of FPDI. The psychometric properties and internal consistency of the questionnaire indicate the validity and reliability of this tool.

## Introduction

An invasive procedure is one where purposeful/deliberate access to the body is gained via an incision, percutaneous puncture, where instrumentation is used in addition to the puncture needle, or instrumentation via a natural orifice [1]. Family Presence During Invasive Procedures (FPDIP) can be defined as the presence of one or more family members at the site where invasive procedures (IP) are performed and that the family members are able to maintain visual or physical contact with the patient and the healthcare team, analogous to Clark’s definition of Family Presence During Resuscitation and Invasive Procedures (FPDRIP) [2].

The approach to Family Presence During Resuscitation and Invasive Procedures (FPDRIP) was studied repeatedly, bearing in mind various ethical and legal aspects [3], since it has a direct impact on patients, family members and healthcare providers (HCP) [4, 5]. In this sense, different studies showed that, while families opted positively towards FPDRIP and perceived it as a right, especially if it occurred in minors [6–8], HCPs presented a greater diversity of opinions in this regard based on their experience, their professional training in dealing with critical patients, or the institutional policy in force at their place of work [7–10].

Gender, profession, culture of origin, religion, level of education and years of experience are the influential variables, according to the evidence, on the attitudes of HCP in relation to PFDRIP. Thus, it is identified that expert nurses manifest a greater predisposition towards FPDRIP [11–14].

The main reasons that support the presence of family members during resuscitation (FPDR) focus on providing more reassurance and calm to the patient during resuscitation, and that family members receive more information about the situation [15–18]. On the contrary, the risk that the family would interfere negatively during the procedures or that the visualisation of the procedures would generate greater anguish, were the reasons to justify that this was not the case [15, 16, 19, 20]. People with a better economic position, older people, chronic patients or those with serious illnesses are those who offer the greatest support for the FPDR [21].

If we focus on the study of Family Presence During Invasive Procedures (FPDIP), it is identified that the willingness to accompany is influenced by the courage and the knowledge of the accompanying persons, and by the degree of invasiveness of the procedures performed [22, 23]. The absence of such accompaniment results in feelings of sadness, despair, helplessness and a feeling of family abandonment experimented by family members [24]. In addition, patients’ preferences in this regard should be taken into account, although there is no direct evidence of reducing their level of anxiety and pain perception during medical or nursing interventions [25].

For their part, many physicians resist the FPDIP due to concerns related to the training of other HCP in critical situations, the medico-legal implications, the possibility of reducing the quality of care caused by distractions or alterations of the sterile environment, despite of the existence of limited evidence related to these factors [26]. That is why many of these HCP indicate the need to develop guidelines, consensual protocols and educational programs that guide clinical practice in these sensitive situations [11, 16, 27].

Curiously, many of the invasive procedures performed on patients at home are witnessed by their relatives [28]; while these, if they are carried out in a hospital environment, imply their exclusion. Family presence allows to provide the sick person with emotional care that HCP cannot substitute [29]. The comprehensive care of the patient is forgotten and the relatives’ desire to remain “by the side” is ignored. In addition, a valuable opportunity to train family members in relation to the care that the patient should receive on their return home is lost [30].

The purpose of this study is to know the attitudes and beliefs of HCP in relation to Family Presence During Invasive Procedures (FPDIP) through a new instrument, originally designed by Twibell et al. [31], to deepen the knowledge about family presence during resuscitation. This instrument was translated cross-culturally and modified in order to learn the opinions, perceptions and experiences of HCP in relation to the FPDIP. A cross-sectional pilot study will be conducted, and the effectiveness of the new instrument will be tested.

## Methodology

### Study tool design

The Twibell et al. [31] questionnaire was selected and translated into Spanish, where the opinions, experiences, and perceptions of the medical and nursing staff about resuscitation witnessed by the family were explored [32]. This tool was modified to probe the same parameters, but in relation to invasive procedures (IP). In turn, due to the degree of invasiveness of the procedures, they were divided into high, medium or low complexity according to the Guidelines for Performance of Invasive Procedures by Medical Students of Yale University School of Medicine [33]. The new tool consisted of three questionnaires related to FPDIP, a socio-demographic one, a Risk-Benefit assessment questionnaire (FPRB) and a Self-Confidence evaluation questionnaire (FPSC). The FPRB scale consisted of 22 items with 5 points on the Likert scale and ranged from strongly disagree (1) to strongly agree (5). A high score indicated a high level of perceived benefit from the FPDIP. The FPSC consisted of 15 items on self-confidence, from not being confident (1) to very confident (5). A high score indicated a high level of self-confidence to handle the FPDIP. Subsequently, a previous pilot test was carried out to evaluate the understanding of the questionnaire questions by 25 members of the health personnel, and a Delphi group made up of experts in different subjects was convened and a final draft was reached. Finally, an item with understanding difficulties was identified; item 9 was changed and items 29 and 30 were removed because they did not proceed in the IP (See Table 1).

**Table 1:** Table of amendment questions. Questionnaire for IP

FPRB-FPSC for RCP	Convert IP. Modifications.
9.-Family members who witness a failed resuscitation will have a better and healthier grieving process	9.- Family members who witness a failed invasive procedure will have a better understanding of what happened.
29.- I could perform electrical therapies during resuscitation efforts with family members present.	Removed
30.- I could deliver chest compressions during resuscitation efforts with family members present	Removed

### Population, sample and data collection

The 541 professionals who are part of the medical and nursing staff of the Consorci Sanitari del Garraf (CSG) were invited via corporate email to participate. There were 344 nurses and 197 physicians.

In the email, they were informed of the characteristics of the study and sent the link to Google forms so that they could answer online. The survey was also distributed in paper format by the different services of the CSG, and a box was provided for the collection of completed questionnaires. The data collection period was from September 1, 2019, to November 30, 2019.

### Data analysis

To measure the performance of the instrument used, a correlation analysis of the items and the scale as a whole (item-test correlation) was performed. The retention criterion for each item was that the correlation was significant, following the indications of Crocker and Algina [34]. Cronbach’s alpha was calculated to determine the reliability of the scale [35]. The calculation of the relationship between the scores of the Perceived Benefit and Risk scales and Self-confidence was calculated with Spearman’s r correlation (rs). To determine the relationship between demographic and perceptual variables, non-parametric tests were used given the nature of the variables (ordinal) and the sizes of the sub-samples. Next, statistical tests such as the Mann-Whiney U (Z) were used to contrast the differences between the two groups and the Kruskal Wallis Test (similar to ANOVA), to contrast more than two groups.

## Results

### Instrument reliability

To assess the reliability of the scales, an item-total analysis was performed. Tables 2 and 3 show the results of applying the maximum likelihood method with varimax rotation. The correlations indicate how each variable contributes to that dimension and is expressed through the Pearson correlation coefficient. High values of the coefficient (positive or negative) indicate that this certain variable contributes significantly to the creation of said dimension. When carried out on the Risk-Benefit scale, it was determined that items 3, 5, 12, 13 and 14 presented a low interrelation with the rest of the items on the scale, so they were removed from the questionnaire. The Cronbach’s alpha value of the scale after removing the five questions is 0.877. When performing the reliability analysis on the Self-Confidence Index scale before the FPDIP, all items showed a correlation with the total greater than 0.5, so no item was removed, and in this case, Cronbach’s alpha was 0.937 (See Tables 2 and 3).

**Table 2:** Perceptions of Self-Confidence in FPDIP

Item number (original order)/Loads (%)		F1 44.66	F2 10.99	F3 7.24
27	Could inform family members present during the development of invasive techniques		**0.717**	
28	I could administer medication during a IPs witnessed by relatives		**0.734**	
31	I could communicate effectively with the rest of the healthcare team during an invasive process witnessed by relatives		**0.765**	
32	Could maintain the patient’s dignity during an IP witnessed by relatives		**0.735**	
33	It could identify family members who have appropriate behaviors during IP	**0.569**		
34	You may be able to prepare family members to access the room where your loved one’s IP is being performed.	**0.764**		
35	You may be able to get the physicians/nurses caring for you to support family presence during your loved one’s IP.	**0.696**		
36	You could accompany family members who are witnessing their loved one’s IP technique.	**0.823**		
37	You could inform the healthcare team that the IP is being witnessed by relatives.	**0.747**		
38	May provide comfort measures to family members present during your loved one’s IPs	**0.785**		
39	You may be able to identify the spiritual and emotional needs of family members present during your loved one’s IPs.	**0.773**		
40	May encourage family members to talk with their loved one during IPs	**0.731**		
41	You may delegate duties to other nurses/physicians to support family members present during your loved one’s IPs	**0.574**		
42	You could inform family members after performing invasive procedural techniques:			**0.813**
43	I could coordinate bereavement follow-up for family members after an IP if needed.			**0.870**
44	You would like your family members to be present while you are undergoing an IP		**0.538**	

Extraction method: principal component analysis

Rotation method: Varimax with Kaiser normalization a

The rotation has converged in 6 iterations

KMO and Bartlett test Kaiser-Meyer-Olkin measure of sampling adequacy 0.874

Bartlett’s sphericity test Approx: Chi-square 1064.582; gl 120; Sig 0.000

**Table 3:** Perceptions of Risk-Benefit in FPDIP

Item number (original order)/Loads (%)		F1 43.60	F2 8.40	F3. 6.60
1	Family members should be given the option to be present while their loved one is undergoing an IP		**0.658**	
2	Family members would be terrified to witness the technique of an invasive procedure		**−0.661**	
4	The health team could develop a close relationship with relatives who witness IP compared to relatives who do not witness them		**0.480**	
6	Family members should be given the option to be present while their loved one is undergoing an invasive procedure		**0.673**	
7	Patients do NOT want their family members to be present during IP			
8	The healthcare team would work harder if family members were present during IP		**−0.560**	
9	Family members who witness a failed invasive procedure will have a better understanding of what happened			**0.536**
10	If my loved one is having an invasive procedure, I should be allowed to be present as I am a nurse or physician			**0.539**
11	The presence of family members will interfere with the techniques of IP	**−0.491**		
15	Family members in the unit where I work prefer to be present during IP		**0.498**	
16	FPDIP is beneficial for patients		**0.505**	
17	FPDIP is beneficial for families		**0.493**	
18	FPDIP is beneficial for nurses	**0.623**		
19	FPDIP is beneficial for physicians	**0.598**		
20	FPDIP should be part of family centered care	**0.568**		
21	FPDIP will have a positive effect on the patient’s hospital care satisfaction survey	**0.845**		
22	FPDIP will have a positive effect on the satisfaction survey of the hospital by the family	**0.844**		
23	FPDIP will have a positive effect on the nurse’s hospital care satisfaction survey	**0.820**		
24	FPDIP will have a positive effect on the physician’s hospital care satisfaction survey	**0.812**		
25	The presence of family members during IP is a right that all patients should have			**0.723**
26	The presence of family members during IP is a right that all family members should have.			**0.714**

Extraction method: principal component analysis

Rotation method: Varimax with Kaiser normalization a

The rotation has converged in 9 iterations

KMO and Bartlett test Kaiser-Meyer-Olkin measure of sampling adequacy 0.862

Bartlett’s sphericity test Approx: Chi square 1753.463; gl 210; Sig 0.000

The risk and benefit index in the FPDIP had a mean score of 3.13 out of 5, with a standard deviation (SD) of ±0.52, while, for self-confidence, it had a mean of 3.32 with a SD ±0.73. The range in RB ranges from a minimum score of 1.71 to a maximum of 4.39 and that of SC was between 1 and 1.48.

The resulting F2 dimension (Table 2) correlates strongly with variables that measure information about the patient’s relatives positively. Items 27, 28, 31 and 32 present r > 0.70. Item 4, with a lower correlation, is associated with this dimension since it directly involves family members (in this case, the person who answers the questionnaire). The F1 dimension includes the correlation with the variables that collect a direct interaction that has to do with the procedures. Also, most of the correlations are r > 0.70. And dimension F3 covers two variables that focus on the period after the intervention, as a conclusion of it concerning families. The correlations of items 42 and 43 with this dimension are r > 0.80.

Regarding Table 3, weaker correlations are observed between the dimensions and the variables that compose them. Dimension F2 is mainly made up of variables related to the exercise of the rights of family members to witness the intervention, as well as the benefits that it would bring to both family members and patients. However, the correlations here are between r|0.40 < r < 0.70|.There is a clearer correlation among the items of the F1 dimension: most of them refer to the positive consequences that the presence of relatives has on the performance of the professionals and their job satisfaction. And finally, dimension F3 is strongly correlated with the items that expose the right that both patients and their relatives must be present (r > 0.70).

### Sample results

Of the 120 professionals who responded to the survey, representing 22.18% of the population of medical and nursing personnel, 77.5% (*n* = 93) of them were women. 41.7% (*n* = 50) were between 36 and 46 years old, and only 19.5% (*n* = 24) were under 35 years of age. 63.3% (*n* = 76) of participants were nursing professionals and the work units with the most significant number of participants were the acute hospital ward with 33.3% (*n* = 40); socio-health centre, with 21.7% (*n* = 26) and the emergency department, with 12.5% *n* = 15). Regarding their training, 46.7% (*n* = 56) were graduates and more than half of the sample, 51.7% (*n* = 62) included a training speciality. It is relevant that the sample was composed mainly of professionals who worked in the acute care, social health and emergency units, so that it can be deduced that the answers obtained are the result of their own experiences, since in their professional areas IP is carried out frequently (See Table 4).

**Table 4:** Sociodemographic variables

Questions	Variables	Frec	%	Questions	Variables	Frec	%
**Gender**	Female	93	77.5	**Professional Status**	Nurse	76	63.3
	Male	27	22.5		Physician	42	35
**Age (years)**	From 20 to 25	3	2.5	**Highest Level of education completed**	Graduate/Bac	56	46.7
	From 26 to 35	21	17.5		Postgraduate	1	0.8
	From 36 to 45	50	41.7		Master	46	10
	From 46 to 55	26	21.7		PhD	12	10
	More than 55	20	16.7		Others	5	4.16
**Unit where you work most often**	Emergencies	15	12.5	**Experience (Years)**	Less than 1 year	4	3.3
	ICU	4	3.3		From 1 to 5	15	12.5
	Social Healthcare	26	21.7		From 6 to 10	12	10
	Acute Hospital	40	33.3		From 11 to 15	24	20
	Residency	2	1.7		From 16 to 20	20	16.7
	External consultation	8	6.7		More than 20	45	37.5
	Day centre/hospital	1	8	**Professional speciality**	YES	62	51.7
	Others	24	20		NO	58	48.3

Only 45% (*n* = 54) of the sample invited a relative to witness an IP carried out on a family member, and of these, only 19.2% (*n* = 23) did it more than five times, there is a relationship between the fact that the professional was previously invited to FPDIP with the number of times the professional invited (X2 = 20.80; *p* < 0.001). In general, there is no association between years of experience and the number of times they allowed the FPDIP, highlighting that the majority (41%) of those who facilitated the FPDIP had 11 to 15 years of experience. On the other hand, there is a relationship between those who show greater self-confidence with expressing the desire for FPDIP in the Advance Care Planning.

There is a significant difference between the perceptions of the FPRB (F 3.67, *p* = 0.01) and FPSC (F 6.31, p < 0.001) indices and the opinion of who should decide on the FPDIP. The participants who presented a higher confidence index (Mean score: 3.6) considered that the person responsible for making the decision was the patient (44.2%; *n* = 53) and in contrast, those who presented lower Self-Confidence (Mean score: 2.9) indicated that the physician (34.2%) was responsible for the invitation. A similar result was obtained with the Risk-Benefit scale. Those participants who associated the invitation to a more significant benefit (Mean score: 3.26) considered that the decision had to be made by the patient, while those who identified a greater risk with the presence of family members (Mean score: 2.89) considered that the physician was the one supposed to invite to the FPDIP.

The survey questions from 44 to 50 were directly related to personal opinion and experience about the FPDIP. Most of the responses reported that they would not agree for a family member to be present while they were undergoing an IP, being more reluctant those who worked in emergencies. Additionally, it was found that regarding inviting the family, there is no significant difference between genders or work units, but there is a difference between the professions, physicians and nurses, according to the Mann-Whiney U (See Table 5).

**Table 5:** Mann-Whitney U

	**Gender**			
	Female (*n* = 93)	Male (*n* = 27)	Mann-Whiney U (Z)	*p* value
RB low risk	3.1009	3.141	−0.201	0.841
RB moderate risk	3.0376	3.0926	−0.327	0.744
RB high risk	2.9926	3.0399	−0.091	0.927
	**Professional Status**			
	Nursing (*n* = 76)	Physicians (*n* = 42)	Mann-Whiney U (Z)	*p* value
RB low risk	3.1847	2.9386	−2.114	0.034*
RB moderate risk	3.1301	2.8709	−2.286	0.022*
RB high risk	3.0653	2.859	−2.005	0.045*
**How many times have you invited a family member to be present during a resuscitation?**				
	Never (*n* = 66)	Sometimes (*n* = 54)	Mann-Whiney U (Z)	*p* value
RB low risk	3.021	3.2187	−1.895	0.058
RB moderate risk	2.9569	3.1638	−1.913	0.056
RB high risk	2.9289	3.094	−1.501	0.133
**In which unit do you work most often?**				
	Emergency/ICU (*n* = 19)	Others (*n* = 101)	Mann-Whiney U (Z)	*p* value
RB low risk	2.9919	3.1321	−1.007	0.314
RB moderate risk	2.919	3.0746	−1.032	0.302
RB high risk	2.8583	3.0305	−1.205	0.228
**Have you ever been present in the room during IP of one of your family members?**				
	YES	NO	Mann-Whiney U (Z)	*p* value
RB low risk	3.1937	3.0414	−1.435	0.151
RB moderate risk	3.146	2.9714	−1.636	0.102
RB high risk	3.0712	2.9476	−1.077	0.282

* Significance with CN95%

### Relationship measures between variables

The correlation between the Risk Benefit and Self-confidence variables is significant and with a moderate intensity of the relationship (Spearman’s rho of 0.58 and alpha < 0.001). In other words, there is a significant relationship although the strength of the association is moderate. In this case, as in the Twibell article, the perception of greater benefit is related to the perception of greater confidence of the professionals.

There is a significant relationship between the profession and the Risk Benefit (F = 6.10; mean = 1.49; *p* = 0.003) and self-confidence (F = 10.64; mean = 3.32; *p* < 0.001) and a greater perception of benefit (mean 3.48) than the medical group (mean 2.93), as well as greater self-confidence (mean 3.48) than physicians (mean 2.98).

There is no association between the FPRB and FPSC indices and the variables of gender, workplace, and professional experience. A trend is observed along with generational change, since younger professionals are more likely to allow PFDIP.

## Discussion

As can be seen in the results, the first part of the objective is fulfilled, by determining the validity and reliability of the instrument, resulting in adequate levels in its measurements, causing this tool to be used in subsequent studies. Cronbach’s Alpha values indicate good internal consistency, but this instrument’s items refer to the measured construct. The indices extracted in the application of this tool are conditioned to the sample. They may be related to other studies that use different evaluation instruments, as we comment in the following lines.

Regarding the second part of the objective, in the analysis of the sample, the results indicate that nursing professionals are more in favour of FPDIP than physicians, coinciding with the studies of Sim [10] and Al Mutair [11], but contrasting with the research of Abuzeyad [36], in which FPDIP was less accepted by nurses than physicians. This study also details the concern on the part of the healthcare team regarding problems of confidentiality and intimacy when allowing FPDIP, an aspect that contrasts with Albarran [37] where the family members did not allege concerns in this regard.

The FPDIP is supported by the professionals as long as the techniques performed are not complex in terms of invasiveness, like in the studies of Al Mutair. In other words, the acceptance of the FPDIP is inversely proportional to the complexity of the IP, as in the conclusions of the Rodríguez-Vico [23] publication. Most respondents said they themselves would want the option to be with a family member undergoing an invasive procedure or a resuscitation event, and if they were the patient, they would want the option to have their own family present during invasive procedures, results that coincide with those of the Magowan&Melvy [28] study.

A trend is observed along with generational change, since younger professionals are more likely to allow FPDIP, this coincides with the Gorete study, where she quotes Mekitarian & Angelo referring that “the young health professionals considering the family as an active participant in the choice of treatment in any situation is a recent one. The discussion about the autonomy of patients and families when facing therapeutic options was introduced at the undergraduate level in health sciences and medical residency and specialization curricula in recent years [38].

Regarding the subject of decision-making, in this study, it is the patient who is defined as the person to decide whether to allow FPDIP, while other studies, such as Chapman [39] or Hayajneh [27] designate the physician as the person responsible for making this decision.

There are significant predictors in Ellison’s [40] studies, such as training, speciality, and the work area, which contrast with this research, where said predictors are not defined.

Regarding previous experience, this study found a relation between inviting and being invited, although in the literature there is no evidence of such relation, as detailed in Leung’s study [20].

This is the first study at CSG to elicit written data about the decision-making related to family presence during invasive procedures of healthcare professionals and to compare findings for physicians and nurses. The study allows its respondents to reflect on the possibility of permitting the FPDIP.

## Conclusions

The psychometric properties and internal consistency of the questionnaire indicate high validity and reliability, although it is necessary to replicate this study to guarantee the validity and reliability of the tool.

The FPDIP also generates controversy since it alters the routines of health professionals, when considering whether to allow it or not. A trend is observed along with generational change, since younger professionals are more likely to allow FPDIP. In general, CSG health professionals are not in favour of FPDIP, with nurses being most in favour. Health workers who perceive fewer risks and more benefits in FPDIP and have greater self-confidence are more in favour of FPDIP.

### Limitations

This study is a cross-sectional pilot test, but it has had a very low response rate (22.18%). The causes are unknown, it may be due to the long length of the questionnaire and its subsequent abandonment or discomfort and disagreement with the subject, for example.

There may be a gender bias, as only 27 men responded to the questionnaire, compared to 93 women.

There is no evidence enabling to compare the performance of this tool in other studies**.**

### Implications for clinical practice

This scientific article is the first step to explore the subject of FPDIP in adults, generating a tool that enables comparison with other evidence, from a health perspective and in Spanish language. More studies are needed on FPDIP.

## Data Availability

The data sets used and/or analyzed during this study are not yet available, because it is intended to carry out another subsequent study, with the same characteristics, but the main author will provide these data upon request. Please contact at the following email: evamariade.mingo@urv.cat.
